# The efficacy and safety of doravirine/lamivudine/tenofovir disoproxil fumarate in treatment-naïve and treatment-experienced patients with HIV

**DOI:** 10.1080/07853890.2025.2564286

**Published:** 2025-10-11

**Authors:** Honghong Yang, Mei Li, Qian Liu, Qin Zeng, Jing Yuan

**Affiliations:** Division of Infectious Diseases, Chongqing Public Health Medical Center, Chongqing, China

**Keywords:** HIV, doravirine, efficacy, safety

## Abstract

**Background:**

Doravirine (DOR) has demonstrated good efficacy for the treatment of people with HIV (PWH); however, there is limited real-world research in developing countries.

**Methods:**

We retrospectively assessed the efficacy and safety of DOR/lamivudine (3TC)/tenofovir disoproxil fumarate (TDF) at 24 and 48 weeks in treatment-naïve and treatment-experienced PWH.

**Results:**

A total of 83 PWH were included from January 1, 2022, to December 31, 2023. The median age was 40 years (32–54). Twenty-seven patients (32.5%) were treatment-naïve PWH, and 56 patients (67.5%) were treatment-experienced PWH, the most common switch was from integrase inhibitors (37/56) to DOR, followed by efavirenz (18/56) and nevirapine (1/56). In treatment-naïve PWH, the median CD4+ T-cell count was 222.9 ± 144.2 cells/mL at baseline, which increased to 337.8 ± 189.6 cells/μL at week 24 (*p* < 0.001) and to 431.6 ± 259.9 cells/μL at week 48 (*p* < 0.001). The overall VS rate was 76.9% (20/26) at week 24 and 93.3% (14/15) at week 48. Creatinine (Cr) significantly increased from baseline to week 48 (*p* = 0.013) but remained normal. There were no significant differences observed in BMI, glucose, estimated glomerular filtration rate (eGFR), liver enzymes, or plasma lipid levels between the baseline and follow-up data. In treatment-experienced PWH, there were no significant changes in the VS rate, CD4+ T-cell count, Cr, eGFR or liver enzymes between the baseline and follow-up data. However, compared to baseline, statistically significant reductions in plasma lipids were observed at week 24 and at week 48. There was also a significant decrease observed in BMI at week 48 compared with baseline. In addition, anxiety, depression and sleep disorders improved in those patients who switched regimens from efavirenz to DOR.

**Conclusions:**

We provide a short-term observational report of the efficacy and safety of DOR/3TC/TDF in routine clinical practice, further supporting its use in PWH.

## Introduction

Since the introduction of combined antiretroviral therapy (ART) in 1996 (which is able to vastly improve life expectancy), human immunodeficiency virus (HIV) has transformed from a fatal condition to a manageable chronic disease [1]. The increased longevity associated with HIV therapy indicates that patients will require decades of ART. However, adverse events that may lead to treatment discontinuation include neuropsychiatric toxicity, skin rashes, abnormal serum lipid levels, abnormal renal or bone parameters, and drug-drug interactions. Moreover, newer ART drugs exhibiting fewer unwanted side effects, including the integrase inhibitor (INSTI), part of boosted protease inhibitor (PI) and novel nonnucleoside reverse-transcriptase inhibitor (NNRTI), have been developed.

Doravirine (DOR) is a novel NNRTI that has demonstrated good efficacy for the treatment of people with HIV (PWH) [2,3]. DOR can be taken without regard for food restrictions [4] and exhibits a low potential for drug-drug interactions [5]. DOR also exhibits a unique resistance profile, possesses a low rate of resistance, and is active against mutations commonly associated with other NNRTIs [6]. In a previous study conducted in treatment-naïve PWH, DOR demonstrated comparable efficacy to efavirenz (EFV) and had a favorable safety profile, with lower rates of drug-related adverse events and central nervous system (CNS) events being observed compared to EFV [7]. In another study, DOR demonstrated noninferior efficacy and a superior lipid profile compared with darunavir-ritonavir after 48 weeks [8].

DOR- and INSTI-based ARTs are approved for adults in many countries throughout the world and are recommended in international HIV guidelines, both as first-line regimens and as switch regimens for those exhibiting virologic suppression (VS) [9–11]. However, concerns regarding the INSTI drug class have emerged. INSTI-based ART has been associated with increases in body weight, changes in lipid profiles, new cardiovascular events and new incidences of hypertension [12–14]. DOR is recommended for the treatment of HIV-1 infections in adults without resistance to NNRTIs or lamivudine (3TC) or tenofovir disoproxil fumarate (TDF) [11], it may represent an alternative to INSTI or older types of NNRTI when considering the above mentioned side effects. Studies in developed countries (e.g. the Netherlands, Italy) have demonstrated the benefits of DOR in maintaining viral suppression, CD4 count recovery, and metabolic health [15,16]. Although similar outcomes have been observed in developing countries [17], research data from developing countries remain relatively scarce.

Chongqing, as a significant regional hub in southwestern China, plays a critical role as an economic, transportation, and manufacturing center. Its strategic location along the upper Yangtze River and its status as a key node in the Belt and Road Initiative further enhance its importance in driving regional development. Chongqing is more heavily affected by the HIV epidemic compared with other regions in China. Chongqing Public Health Medical Center (CPHMC) is the only tertiary infectious disease specialized hospital in Chongqing. Specifically, this hospital is designated to treat PWH and serves as a quality control center for HIV diagnosis and treatment in the region. As a quality control center, CPHMC sets clinical standards, supervises HIV treatment protocols, ensures quality across healthcare facilities in the region. Here, we report the efficacy and safety of 48 weeks of DOR/3TC/TDF treatment in treatment-naïve PWH at the CPHMC. We also evaluated the efficacy and safety of switching to 3TC/TDF/DOR from a stable ART regimen in treatment-experienced PWH.

## Methods

### Patients

Data were collected from all HIV patients who started ART regimens with DOR (100 mg)/3TC (300 mg)/TDF (300 mg) and those who switched to DOR/3TC/TDF when they achieved virological suppression for ≥ 6 months at the CPHMC from January 1, 2022, to December 31, 2023. The inclusion criteria were 1) having a diagnosis of HIV infection; 2) aged ≥ 18 years; 3) designated as treatment-naïve PWH who have never received ART, or treatment-experienced PWH who have received ART with VS (HIV-RNA viral load (VL) <50 copies/mL for at least 6 months); and 4) without documented genotypic resistance to any of the components of DOR/3TC/TDF, genotypic resistance testing was performed at baseline (first ART initiation) using RNA-based assays, HIV-1 drug resistance were analyzed using the HIV-1 pol sequence. The exclusion criteria were as follows: 1) being pregnant; 2) Child-Pugh Score Grade C of liver function; and 3) exhibiting renal failure with creatinine clearance of <50 mL/min.

Venous blood was collected *via* an EDTA anticoagulant tube and centrifuged to collect the plasma. The plasma was then frozen at −80 °C until use. HIV-RNA VL was quantified by the COBAS Ampliprep/TaqMan 48 real-time RT-PCR test (Roche, Germany) according to the manufacturer’s instructions, with a lower limit of quantification of 20 RNA copies per milliliter. The CD4+ T-cell count was assessed with a BD FACSCanto II flow cytometer (BD Biosciences, USA). All clinical laboratory tests were performed following an 8- to 12-h overnight fast.

The physician will determine an appropriate antiretroviral therapy (ART) regimen in accordance with the Chinese Guidelines for HIV/AIDS Diagnosis and Treatment and based on the patient’s baseline and follow-up laboratory results. Following the provision of informed consent, the patient will initiate the 3TC/TDF/DOR regimen.

### Data and information collection

Data on baseline demographic and clinical characteristics were collected, including sex, age at HIV diagnosis, transmission route, presence of HBV/HCV co-infection, and pre-existing chronic disease (diabetes and hypertension). Laboratory results such as CD4+ T-cell count, HIV-RNA VL, alanine aminotransferase (ALT), aspartate aminotransferase (AST), total cholesterol (TC), triglycerides (TG), low-density lipoprotein cholesterol (LDL-c), high-density lipoprotein cholesterol (HDL-c), estimated glomerular filtration rate (eGFR) and Cr were obtained. These indicators were collected at baseline and each follow-up visit (week 24 and week 48). In addition, levels of anxiety and depression were assessed *via* the Hamilton Anxiety Scale (HAMA) and Montgomery-Asberg Depression Rating Scale (MADRS); moreover, sleep quality was assessed using the Pittsburgh Sleep Quality Index (PSQI).

### Outcomes

Our primary objective was to evaluate the VS rates and immunologic responses in treatment-naïve and treatment-experienced PWH. Our secondary objective was to evaluate treatment safety (including variations in body mass index (BMI); glucose, lipid, and liver enzyme levels; kidney function; and neuropsychiatric conditions) from baseline to week 48.

### Statistical analysis

All of the study data were analyzed using IBM-SPSS Statistics software, version 25.0 (IBM-SPSS Statistics, Armonk, New York, USA). Standard descriptive statistics were used to analyze the demographic and clinical characteristics, as well as the laboratory results of the patients. Continuous variables with a normal distribution are reported as the means ± standard deviations (SDs), whereas continuous variables with a nonnormal distribution are presented as medians with interquartile ranges. Changes in the continuous variables with a normal distribution were tested *via* the paired t test from weeks 0 to 48 in the same group, whereas for continuous variables with a nonnormal distribution, changes were tested *via* the paired Wilcoxon test. Categorical variables were compared using either chi-square tests or Fisher’s exact tests. A *p*-value <0.05 was considered to indicate statistical significance.

## Results

### Study population

A total of 101 eligible cases were initially included; after excluding patients without available data and those lost to follow-up, a total of 83 patients were enrolled in the study. Among the 18 excluded patients, 7 were transferred to local healthcare facilities for ART and were not followed up further at our center, while the remaining 11 switched back to the national free treatment regimen within 12 months due to financial constraints. None of the exclusions were attributable to adverse reactions to DOR. Among the 83 included patients, the median age was 40 years (interquartile range [IQR]: 32–54), and 81.9% (68/83) of the patients were male. Heterosexual transmission was the primary route of HIV transmission, accounting for 60.3% (50/83) of the cases. Common comorbidities were hypertension (*n* = 5) and diabetes (*n* = 2), with one patients had both conditions. The coinfections included syphilis (*n* = 2), with no patients of concurrent hepatitis B virus (HBV) or hepatitis C virus (HCV) infection. In total, 27 patients (32.5%) were classified as treatment-naïve PWH, and 56 patients (67.5%) were classified as treatment-experienced PWH.

### Virologic outcomes and immunologic responses in treatment-naïve PWH

At baseline, the median CD4+ T-cell count of the 27 ART-naïve patients was 222.9 ± 144.2 cells/mL; moreover, with 44.4% (12/27) of patients exhibited HIV-RNA VL> 100,000 copies/ml. Follow-up data demonstrated significant improvement: by 24 weeks, 76.9% (20/26) of PWH achieved VS with a significant increase in median CD4+ T-cell count to 337.8 ± 189.6 cells/µL (*p* < 0.001 vs baseline). Outcomes further improved at week 48, showing a 93.3% (14/15) VS rate and a median CD4+ T-cell count rising to 431.6 ± 259.9 cells/µL (*p* = 0.004) (Table 1).

**Table 1.** Virologic outcomes and immunologic responses at weeks 24 and 48.

Characteristics	Baseline	Week 24	Week 48
CD4+ T-cell counts in treatment-naïve PWH (cells/mL)	222.9 ± 144.2	337.8 ± 189.6	431.6 ± 259.9
*p*-value^a^		<0.001	0.004
CD4+ T-cell counts in treatment-experienced PWH(cells/mL)	523.2 ± 255.5	535.7 ± 315.9	486.1 ± 321.9
*p*-value^a^		0.426	0.844
HIV-RNA < 50 copies/mL in treatment-naïve PWH n (%)	27 (0%)	76.9% (20/26)	93.3% (14/15)*
HIV-RNA < 50 copies/mL in treatment-experienced PWH n (%)	100% (56/56)	100% (54/54)	98.2% (54/55)

^a^ *p*-value indicating the significance of the change from baseline to the follow-up time point.

* 12 participants with vs at week 24 declined subsequent testing at week 48 due to economic and personal reasons.

### Virologic outcomes and immunologic responses in treatment-experienced PWH

The 56 treatment-experienced PWH, including individuals receiving bictegravir, emtricitabine and tenofovir alafenamide (BIC/FTC/TAF) (48.2%, 27/56); 3TC/TDF/EFV (efavirenz) (30.4%, 17/56); 3TC/dolutegravir (DTG) (8.9%, 5/56); elvitegravir (8.9%, 5/56); 3TC/AZT (azidothymidine)/EFV (1.8%, 1/56); and 3TC/AZT/NVP (nevirapine) (1.8%, 1/56), achieved VS ≥ 6 months before the ART regimens were changed to DOR/3TC/TDF, and the median CD4+ T-cell count was 523.2 ± 255.5 cells/μL at baseline. Post-transition outcomes demonstrated good virological control, with 100% VS (54/54) at 24 weeks and 98.2% VS (54/55) at 48 weeks. Immunological parameters remained stable throughout follow-up, with median CD4+ counts of 535.7 ± 315.9 cells/μL at 24 weeks and 486.1 ± 321.9 cells/μL at 48 weeks, with no statistically significant differences from baseline values observed at either time point (*p* > 0.05).

### Safety and metabolic profile in treatment-naïve PWH

At 24 and 48 weeks, there were no significant differences observed in the eGFRs between the baseline and follow-up data in the treatment-naïve PWH; however, the Cr level significantly increased between baseline and 48 weeks (*p* = 0.013) (Table 2). ALT and AST levels showing no significant differences from baseline at both 24- and 48-week assessments (*p* > 0.05). Metabolic parameters, including glucose, lipid profile (including TC, TG, LDL-c, HDL-c), and BMI similarly maintained stability throughout the study duration, all showing no statistically significant from baseline values (*p* > 0.05).

**Table 2.** Changes in hepatic safety, renal safety and lipid profiles between baseline and follow-up data in treatment-naïve PWH.

Characteristics	Baseline	Week 24	Week 48
BMI (kg/m^2^)	23.8 ± 3.2	–	24.6 ± 2.7
*p*-value^a^		–	0.878
ALT (U/L)	23 (15, 42)	26 (16, 29)	25 (18, 27)
*p*-value^a^		0.400	0.313
AST (U/L)	28 (21, 35)	27 (20, 33)	22 (19, 30)
*p*-value^a^		0.441	0.477
Creatinine (umol/L)	70.5 ± 15.1	75.2 ± 15.6	76.0 ± 16.5
*p*-value^a^		0.128	0.013
Glomerular filtration rate (mL/min)	103.8 ± 19.5	99.6 ± 21.0	100.5 ± 22.3
*p*-value^a^		0.160	0.060
Glucose (mmol/L)	5.64 (5.32, 6.21)	5.80 (5.11, 6.44)	5.53 (5.33, 6.52)
*p*-value^a^		0.840	0.434
Total cholesterol (mmol/L)	3.91 (3.44, 4.27)	3.89 (3.50, 4.49)	3.73 (3.59, 4.25)
*p*-value^a^		0.221	0.670
Triglycerides (mmol/L)	1.43 (1.08, 2.31)	1.50 (0.88, 2.09)	1.49 (1.16, 2.09)
*p*-value^a^		0.223	0.156
LDL cholesterol (mmol/L)	2.13 (1.78, 2.54)	2.30 (1.97, 2.58)	2.18 (1.80, 2.59)
*p*-value^a^		0.077	0.069
HDL cholesterol (mmol/L)	1.00 ± 0.21	1.09 ± 0.22	0.99 ± 0.15
*p*-value^a^		0.077	0.224

^a^ *p*-value indicating the significance of the change from baseline to the follow-up time point.

ALT: alanine aminotransferase; AST: aspartate aminotransferase; LDL: low-density lipoprotein; HDL: high-density lipoprotein.

### Safety and metabolic profile in treatment-experienced PWH

At 24 and 48 weeks, the glucose, renal and hepatic profiles did not significantly change in treatment-experienced PWH (Table 3). Significant improvements in lipid profiles were observed, with median TC decreasing from 4.73 mmol/L (IQR 4.04–5.74) to 4.24 (3.58–5.02) at week 24 (*p* = 0.001) and 4.18 (3.43–4.91) at week 48 (*p* = 0.030). Similar reductions were seen in TG (2.01 to 1.55 mmol/L, *p* = 0.003), LDL-c (2.64 to 2.54 mmol/L, *p* = 0.024), and HDL-c (1.12 to 1.02 mmol/L, *p* = 0.002) at week 24, with sustained LDL-c (2.26 mmol/L, *p* = 0.036) and TG (1.70 mmol/L, *p* = 0.033) benefits at week 48. Notably, participants transitioning from integrase inhibitors (INSTIs) regimens to 3TC/TDF/DOR (*n* = 37) exhibited a significant BMI reduction from 26.1 ± 0.6 to 25.4 ± 0.6 kg/m^2^ (*p* < 0.05). In addition, PWL who switched from integrase inhibitors (INSTIs) regimens to 3TC/TDF/DOR (*n* = 37) exhibited a significant BMI reduction from 26.1 ± 0.6 to 25.4 ± 0.6 kg/m^2^. (*p* < 0.05).

**Table 3.** Changes in hepatic safety, renal safety and lipid profiles between baseline and follow-up data in treatment-experienced PWH.

Characteristics	Baseline	Week 24	Week 48
BMI (kg/m^2^)	26.1 ± 0.6	–	25.4 ± 0.6
*p*-value^a^			0.004
ALT (U/L)	32 (24, 46)	28 (22, 47)	27 (21, 42)
*p*-value^a^		0.417	0.098
AST (U/L)	27 (21, 32)	29 (21, 37)	26 (21, 32)
*p*-value^a^		0.238	0.640
Creatinine (umol/L)	71.9 (61.6, 81.5)	73.9 (62.4, 85.0)	74.9 (60.1, 86.7)
*p*-value^a^		0.534	0.169
Glomerular filtration rate (mL/min)	108.0 (97.0, 118.8)	110.0 (92.0, 118.0)	109.0 (91.8, 115.0)
*p*-value^a^		0.360	0.115
Glucose (mmol/L)	5.76 (5.32, 6.44)	5.64 (5.30, 6.10)	5.60 (5.30, 5.89)
*p*-value^a^		0.471	0.743
Total cholesterol (mmol/L)	4.73 (4.04, 5.74)	4.24 (3.58, 5.02)	4.18 (3.43, 4.91)
*p*-value^a^		0.001	0.030
Triglycerides (mmol/L)	2.01 (1.20, 3.71)	1.55 (1.06, 2.70)	1.7 (1.15, 2.32)
*p*-value^a^		0.003	0.033
LDL cholesterol (mmol/L)	2.64 (2.14, 3.25)	2.54 (1.94, 2.93)	2.26 (1.99, 2.67)
*p*-value^a^		0.024	0.036
HDL cholesterol (mmol/L)	1.12 (0.98, 1.46)	1.02 (0.89, 1.22)	1.05 (0.90, 1.23)
*p*-value^a^		0.002	0.059

^a^ *p*-value indicating the significance of the change from baseline to the follow-up time point.

ALT: alanine aminotransferase; AST: aspartate aminotransferase; LDL: low-density lipoprotein; HDL: high-density lipoprotein.

### Neuropsychological outcomes following switch from 3TC/TDF/EFV to DOR/3TC/TDF

To investigate the changes in neuropsychiatric conditions associated with the switch from 3TC/TDF/EFV to DOR/3TC/TDF, 15 out of 18 PWH on EFV-based regimen completed the neuropsychological assessments (Table 4). Overall, the HAMA, MADRS and PSQ scores significantly decreased from baseline to week 48 (*p* < 0.05).

**Table 4.** Changes in neuropsychiatric conditions between baseline and week 48 after switching from EFV to DOR (*n* = 15).

Characteristics	Baseline	Week 48	*p*
HAMA score	4 (2, 7)	2 (1, 5)	0.003
MADRS score	2 (0, 6)	1 (0, 4)	0.038
PSQ score	5 (4, 10)	4 (3, 6)	0.002

## Discussion

In the context of modern ART, this retrospectively study evaluates the efficacy and safety of DOR/3TC/TDF in both treatment-naïve and treatment-experienced PWH. Our results demonstrated that DOR/3TC/TDF exhibits efficacy in the suppression of HIV-1 replication and an increase in CD4+ T-cell counts in treatment-naïve PWH. We also observed that switching to DOR/3TC/TDF from stable ART regimens (including second-generation INSTIs or NNRTIs) exerted a beneficial effect on lipids and BMI, accompanied by maintained VS and immunologic responses.

In our study, DOR/3TC/TDF is an effective regimen for achieving VS in treatment-naïve PWH. with VS rate of 76.9% at week 24 and increasing to 93.3% at week 48- superior to the 84.3% reported in the DRIVE-AHEAD study [18], potentially attributable to the lower proportion of high baseline viral loads in our study (44.4% vs 81.2% with HIV RNA >100,000 copies/mL, respectively). In addition, during the week 48 follow-up period, durable VS was also observed after switching to DOR/3TC/TDF in treatment-experienced PWH, with the exception of one patient. The results were consistent with previous studies [15,16,19]. An Italian retrospective study showed that the effectiveness of the DOR/3TC/TDF regimen in maintaining vs among PWH on ART, with a low 1-year treatment failure risk [15], and the Athena cohort demonstrated maintained viral suppression at 96 weeks in virologically stable PWH switching to DOR [16]. Current evidence supports switching to DOR/3TC/TDF is a generally well-tolerated option for maintaining viral suppression in adults considering a change in therapy [19].

Immunologic responses, measured by CD4+ T-cell increases, significantly improved at 24 and 48 weeks in treatment-naïve PWH. This result was similar to previous studies. Robust evidence confirms CD4+ T-cell count recovery in treatment-naive patients [6,20]. Specifically, Orkin C et al. reported that the mean increase in the CD4+ T-cell count from baseline to 48 weeks was 195.5 cells/μL for DOR in treatment-naïve PWH [6], and an increase in the CD4+ T-cell count has also been observed in adolescents who used DOR [20]. Among treatment-experienced patients with long-term viral suppression, CD4+ T-cell count remained largely stable after switching regimens to DOR/3TC/TDF. In total, DOR/3TC/TDF can promote and maintain immune reconstitution in PWH.

HIV infection itself and ART contribute to metabolic disturbances, including weight gain, lipid dyslipidemia and glucose metabolism impairment. The newly introduced ARTs, including INSTIs and TAFs, are well known to be related to weight gain [21]. Our findings demonstrate that DOR/3TC/TDF regimens offer distinct metabolic advantages. There was no significant weight gain observed in treatment-naïve PWH initiating DOR, and those patients experienced weight loss when they switched from INSTIs to DOR. These results are similar to those of prior studies [21,22]. DOR seems to be a good option for patients with a high BMI. ART is often associated with lipid profile perturbations and increasing levels of proinflammatory lipid species. These alterations in lipid values (including TC and LDL-c levels) are known to be major risk factors for cardiovascular disease (CVD) [23]. Our results demonstrate that DOR confers beneficial effects on lipid parameters in both treatment-naïve and treatment-experienced patients, consistent with evidence from randomized clinical trials and real-world observational studies [3,24]. The utilization of ART regimens with a low impact on the lipid profile is important for improving quality of life and life expectancy in HIV infection management [25], especially for those patients already at high risk of CVD. TDF has been associated with an independent lipid-lowering effect, and DOR has also been shown to independently improve lipid profiles. Thus, the combination of TDF and DOR may be more beneficial to plasma lipid levels.

Due to the poor economic level and drug accessibility, EFV is still commonly used for first-line ART freely for PWH in China. However, neuropsychiatric events are the most frequently observed side effects of EFV, which is the main reason for switching from EFV to another treatment regimen [26]. In our study, DOR demonstrated a superior neuropsychiatric profile compared with EFV. In addition, renal function seemed to be negatively influenced in treatment-naïve PWH; moreover, Cr significantly increased from baseline to 48 weeks in our study, but was still observed within the normal range. Cr, which represents another measure used to assess renal function, was also observed within the normal range. The elevation of Cr may be related to the use of TDF, and larger populations or longer follow-up periods are needed to assess the kidney effects of this regimen. Overall, the close monitoring of renal function after the initiation of 3TC/TDF/DOR is recommended.

This study has several limitations. Firstly, this study was retrospective in nature and involved a relatively small sample size. Secondarily, it lacked long-term follow-up data, which may introduce some bias in the results. Thirdly, self-adverse feelings cannot be accurately recorded by recall; therefore, drug-related adverse events (AEs) were not thoroughly described in our study. Fourthly, we could not determine the etiology of virological failure due to the unavailability of HIV drug resistance testing. In addition, we assessed neuropsychiatric improvements after EFV-to-DOR switching; the current study design did not capture INSTI-to-DOR conversions - an important area for future research given INSTIs’ dominance in contemporary ART. Despite these limitations, real-life data are particularly needed in developing countries to further evaluate the aforementioned effects.

We provide a short-term observational report of the efficacy and safety of DOR/3TC/TDF in routine clinical practice, further supporting its use in PWH. The DOR/3TC/TDF regimen seems to have the potential to provide health-related quality-of-life benefits; of course, future studies with prospective, larger-scale studies are needed.

## Data Availability

The original data in this study can be obtained from the corresponding author upon reasonable request.
